# A new approach to derive productivity of tropical forests using radar remote sensing measurements

**DOI:** 10.1098/rsos.231186

**Published:** 2023-11-22

**Authors:** Hans Henniger, Andreas Huth, Friedrich J. Bohn

**Affiliations:** ^1^ Department of Ecological Modeling, Helmholtz Centre of Environmental Research (UFZ), Permoserstraße 15, Leipzig 04318, Germany; ^2^ Department of Computational Hydrosystems, Helmholtz Centre of Environmental Research (UFZ), Permoserstraße 15, Leipzig 04318, Germany; ^3^ Institute for Environmental Systems Research, University of Osnabrück, Barbara Straße 12, Osnabrück 49074, Germany; ^4^ iDiv German Centre for Integrative Biodiversity Research Halle-Jena-Leipzig, Puschstraße 4, Leipzig 04103, Germany

**Keywords:** forest model, productivity, biomass, tropical forests, radar, remote sensing

## Abstract

Deriving gross & net primary productivity (GPP & NPP) and carbon turnover time of forests from remote sensing remains challenging. This study presents a novel approach to estimate forest productivity by combining radar remote sensing measurements, machine learning and an individual-based forest model. In this study, we analyse the role of different spatial resolutions on predictions in the context of the Radar BIOMASS mission (by ESA). In our analysis, we use the forest gap model FORMIND in combination with a boosted regression tree (BRT) to explore how spatial biomass distributions can be used to predict GPP, NPP and carbon turnover time (*τ*) at different resolutions. We simulate different spatial biomass resolutions (4 ha, 1 ha and 0.04 ha) in combination with different vertical resolutions (20, 10 and 2 m). Additionally, we analysed the robustness of this approach and applied it to disturbed and mature forests. Disturbed forests have a strong influence on the predictions which leads to high correlations (*R*^2^ > 0.8) at the spatial scale of 4 ha and 1 ha. Increased vertical resolution leads generally to better predictions for productivity (GPP & NPP). Increasing spatial resolution leads to better predictions for mature forests and lower correlations for disturbed forests. Our results emphasize the value of the forthcoming BIOMASS satellite mission and highlight the potential of deriving estimates for forest productivity from information on forest structure. If applied to more and larger areas, the approach might ultimately contribute to a better understanding of forest ecosystems.

## 1. Introduction

Carbon exchanges between the land surface and the atmosphere represent the largest fluxes within the global carbon cycle [1]. These fluxes are mediated by terrestrial ecosystems, where forests play a dominant role in the biosphere–atmosphere interface with evident impacts on climate via biophysical and biogeochemical feedbacks affecting water, carbon, energy fluxes [2]. Therefore, understanding the link between the dynamics of forests (growth, competition, mortality and establishment of new trees) and the dynamics of the carbon cycle from short to long time scales are of fundamental interest and will improve our understanding of possible trajectories under future climate change scenarios [3].

To understand the fluxes related to forest dynamics and also to land use change, biomass is a central driver as it is a key component of the global carbon cycle and therefore identified as an Essential Climate Variable by the United Nations Framework Convention on Climate Change [4]. Linking biomass to carbon fluxes is important to quantify ecosystem services and to develop adapted forest biomass management policies in the context of global warming. In particular, knowledge of biomass in disturbed forests is important because most carbon emissions from land-use change are caused by deforestation.

Knowledge of the regional and global distribution of biomass is limited. Forest inventories are important for better understanding processes of forest dynamics and for calibrating remote sensing measurements. Promising knowledge of biomass distribution over large scales can be obtained from airborne and spaceborne measurements [5,6]. Latest satellite technology leads to increasing progress in quantifying biomass from space (e.g. [7–9]). Technological developments are also leading to advances in the spatial resolution of satellite measurements. At the same time, such developments are associated with additional costs and possible additional error sources. These trade-offs raise the question as to which spatial resolution of satellite measurements are useful to investigate specific forests characteristics. In addition, it should be noted that satellite-based measurements that can map canopy structure or vertical biomass distribution (such as radar) have not yet been used to estimate carbon fluxes, including gross primary production (GPP), net primary production (NPP) and carbon turnover time (*τ*). As first described by Running *et al.* [10], estimates of GPP and NPP currently rely mainly on optical satellite data. However, today’s productivity estimates have the limitation that they cannot provide data when cloud cover is closed.

To better understand the potential of the expected remote sensing data from future satellite missions, we use the well-established forest model FORMIND [11–13]. This model was already used in various studies to better understand the link between remote sensing observations and forest dynamics [14–16]. It can simulate the development of carbon stocks, productivity and carbon fluxes from a single tree up to the whole forests.

With the help of the individual-based Forest Model FORMIND, we will investigate which horizontal and vertical resolutions of remote sensing biomass measurements are best suited to study the link between forest carbon stocks and carbon dynamics of forest, which include (i) GPP, (ii) NPP and carbon turnover time (*τ*). We want to investigate these questions in the context of the P-band radar BIOMASS mission by the European Space Agency. The primary objective of the BIOMASS mission is to determine the distribution of forest above-ground biomass (AGB) worldwide (planned resolution 4 ha) and to reduce the major uncertainties in calculations of carbon stocks using radar space borne observation data.

In our analysis, we (i) introduce an approach where we use the forest gap model FORMIND to better understand the potential of the expected remote sensing data of the BIOMASS and subsequent satellite missions. We (ii) will investigate the relationship between horizontal and vertical structures of AGB and carbon dynamics (GPP, NPP and *τ*) in forests at different spatial scales by using a BRT. We (iii) want to investigate whether our research results are robust to different forest types.

## 2. Methods

We used the individual-based forest gap model FORMIND to simulate the vertical and horizontal biomass distribution over time in an typical tropical forest. These spatial distributions are used to predict GPP, NPP and *τ* of the forest.

### 2.1. FORMIND

FORMIND is an individual-based forest gap model that is used to simulate the growth of forests. In high diversity forests, like in the tropics, species are classified into different plant functional types ([11], www.formind.org). In this study, we have analysed a tropical forest using the parametrization of Knapp *et al.* [16]. The parametrization has been developed for a tropical lowland rainforest (50 ha megaplot, 1000 m × 500 m) on Barro Colorado Island (BCI), Panama (9.15N, 79.85W), which has been continuously monitored for more than three decades. The inventory provides an important source of information for forest model parametrization [16,17] and ground truthing for remote sensing studies [18–20] due to its remarkable spatial and temporal dimensions and the large number of studies associated with it. Thus, the parametrization which was developed for the forest gap model FORMIND has been extensively tested and other sites in South America provide similar results [21].

The model FORMIND simulates the following processes:

— *Establishment*—Seeds are distributed over the forest area. If light conditions are suitable, new trees can establish and compete for light and space.
— *Growth*—The growth of a tree is determined by its GPP, respiration and type-specific physiological parameters.
— *Mortality*—This process is described by a specific mortality rate. If one tree falls, neighbouring trees can be damaged. Additional mortality occurs due to crowding in dense stands or due to low stem diameter increments (stress situations).
— *Competition*—One of the main driving factors of tree growth is light. FORMIND calculates the light condition in different height layers of the forest. A small tree in the shade of a large tree receives less light and therefore reduces its carbon production. A full model description and free code is available at formind.org.

The model calculates the GPP, growth and maintenance respiration, and NPP for each tree. Based on tree mortality, carbon stocks and fluxes between atmosphere, forest stand and soil, the carbon balance of a forest stand can be derived. The vertical and horizontal biomass distribution is an emerging pattern of the simulation.

The parametrization of the forest model for the BCI site was based on data from four 50 ha censuses covering the period 1990–2010 [17]. The analysis showed that the high species diversity of tropical forests (323 tree species at the site) can be represented by four functional plant types (PFTs). This aggregation provides the necessary variability to replicate observed field patterns, while maintaining a manageable model complexity.

Tree height and crown diameter were parametrized based on breast height stem diameter using established allometric equations and measurements from the BCI dataset. Furthermore, a subset of parameters were updated through inverse calibration, where values were varied within appropriate ranges and simulation results were compared with field data to identify new patterns. These calibrated parameters included annual seed growth, background mortality and parameters related to the light response curve for photosynthesis. The calibration was based on metrics such as AGB, basal area, stem number and stem size distribution for each PFT after 500 years of simulation. An optimization algorithm using dynamic dimensional search (DDS) was used in the calibration process [22].

Comparisons between simulated LiDAR data and airborne data showed good agreement in vertical structure. Comparisons of emergent variables from the simulation, such as biomass and stem size distribution, with stand data showed good representation of dynamic processes [16]. The GPP measured at the BCI whirlwind tower is between 52 and 58.8 tonnes per hectare per year [23,24], which is in agreement with our simulation results. The NPP values obtained from the inventory are around 12 tonnes per hectare per year [25]. In summary, the parametrization of our model is well suited for the study of carbon dynamics and forest structure.

Simulation setting: We simulated a total area of 100 ha of a tropical forest over a period of 320 years with a time step of 1 year, consisting of 25 independent simulation runs with a resolution of 200 × 200 m each—which corresponds to the envisaged spatial resolution of the main products of the BIOMASS mission (i.e. AGB). However, the FORMIND model has a spatial resolution of 20 × 20 m, which enables us to analyse finer resolutions.

**Figure 1.**
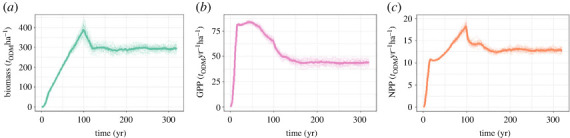
Simulated forest dynamics over time: (*a*) AGB, (*b*) GPP, and (*c*) NPP. Each thin line represents the simulated forest dynamics for a 200 m × 200 m forest plot. The thick lines show the average over all 25 simulations.

The development of the simulated forests covers two phases: the successional phase in which forest grows from a bare ground until they reach the second, the equilibrium phase after 160 years (figure 1).

In the first phase, pioneer species in particular cause a rapid increase in biomass, GPP and NPP. At the peak around the year 100 of the simulation, many of the even-aged large trees of pioneer species die. Afterwards the forest takes over a diverse height structure of trees and a higher diversity in species, which is typical for mature forests which we find in the simulation after 160 years.

### 2.2. Vertical biomass distribution

**Figure 2.**
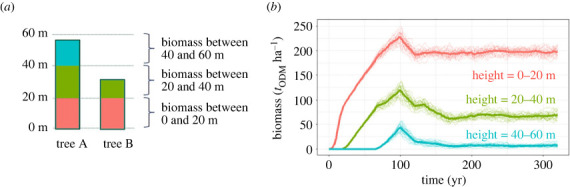
(*a*) Example for the assumed distribution of biomass over height (here height layers of 20 m size) for two trees. The area of the rectangle symbolizes the share of biomass in the corresponding height layer. (*b*) Development of biomass for each 4 ha forest stand (thin lines) at a total simulated area of 100 ha for different height layers Δ*h* = 20 m. The mean biomass for the different height layers are displayed by the corresponding thick lines (different colours corresponding to the different height layers).

In our analysis, we divide the forest into different height layers with a layer size of Δ*h*. To simplify the analysis, we assume here that biomass is equally distributed over the height of a tree [26]. Figure 2*a* shows an example of the biomass distribution in different height layers (Δ*h* = 20 m) of a tree. For the tree A (with a height of 56 m), the same amount of biomass is allocated in the height layer from 0–20 m and 20–40 m (figure 2*a*). Since the tree is not taller than or equal to 60 m, there is less biomass in the 40–60 m height layer. With the help of the forest model, we can analyse the vertical distribution of the biomass over the whole simulation area and investigate the development of biomass in different height layers during forest succession. Figure 2*b* shows the simulated biomass according to the different height layers with a resolution of 200 m × 200 m over time. The higher the height layer is located, the longer it takes for trees to grow into it and the longer it takes to reach the equilibrium phase.

### 2.3. Linking biomass distribution with carbon fluxes

The forest model FORMIND allows us to link the derived biomass with corresponding carbon fluxes. GPP and NPP are derived directly from the model at a spatial resolution of 20 × 20 m as GPP and NPP is calculated for each tree. In this study, we also calculate the *τ*, which can be estimated as follows [27]:

2.1 $$\tau =\frac{\mathrm{biomass}}{\mathrm{GPP}}.$$

The calculation of *τ* is developed for forests in equilibrium state. For exploration we calculate *τ* also for forests which have not reached the equilibrium state.

We developed a framework to calculate GPP, NPP and *τ* in forests at different spatial resolutions. The resolution of biomass information varied both horizontally and vertically. In our analyses, we studied the tropical forest at scales of 200, 100 and 20 m horizontal resolution. In terms of vertical resolution, we have investigated one vertical layer (Δ*h* = 100 m) and layers with a size of Δ*h* = 20 m, which is one possible resolution of the BIOMASS mission, and two additional finer resolutions Δ*h* = 10 m and Δ*h* = 2 m. The simulated forest is the same in all cases (100 ha), only the horizontal and vertical spatial resolution of the extracted biomass distribution and carbon fluxes vary.

We applied BRTs to quantify the predictability of forest productivity using the information of vertical distributed biomass for the three different horizontal resolutions. BRTs are a machine learning algorithm using multiple decision (or regression) trees [28]. Each model was trained in forward stage-wise procedures to predict one target variable (GPP, NPP or *τ*) based on the vertical biomass distribution using cross validation. One part of the forest data were used for training (50% of data but maximal 15 900 datapoints) and the other part was used for the validation (see table 1). We used for the analysis R package dismo 1.3–14 [29].

The BRT uses iterative processes to minimize the squared error between predicted values and those of the dataset. Hereby, part of the data was used for a fitting procedure and the rest was used for computing out-of-sample estimates of the loss function. In order to obtain the best regression model, we analysed all possible combinations of learning rate, bag fraction and tree complexity: for learning rates, we tested 0.05, 0.01, 0.005; for bag fractions, we tested 0.3, 0.5, 0.66; for tree complexity, we chose 3 for the 20 m and 100 m layer widths; we chose 5 for the 10 m layer width; and we tested 5 and 7 for the 2 m layer width case). We assumed a Laplace error structure (the selected parameter combination for the boosted retression trees models can be found in appendix tables 1–3). The obtained best models were used for all further analyses. To determine the best regression model for disturbed and mature forests, we redid the whole training procedure using only data from the equilibrium phase (years 160–320) for mature forests and data before equilibrium phase (0–160 years) for disturbed forests. The best model was then used to predict the mature/disturbed forest data, which were not used for the training.

## 3. Results

### 3.1. Relationships between biomass and GPP, NPP and *τ*

**Figure 3.**
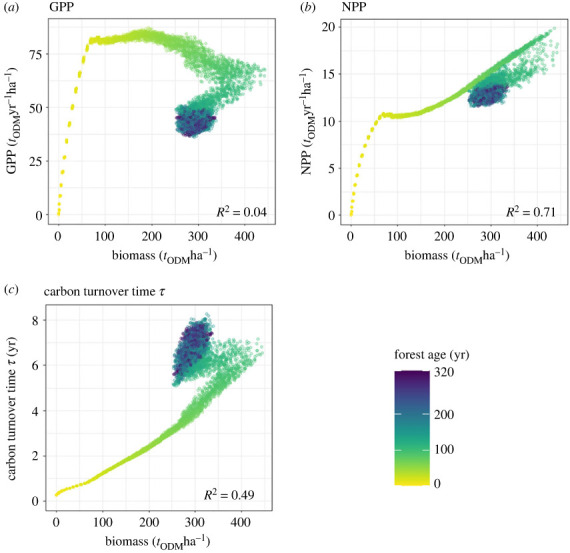
Relationship between biomass and (*a*) GPP, (*b*) NPP and (*c*) *τ*. The results are derived from forest simulations (tropical forest, see §2.1). Each point represents the relationship between biomass and the target variable for a 4-ha forest at a certain age (between 0 and 320 years indicated by colour). Additionally, the *R*^2^ is calculated (right bottom corner of each graph).

Here we analyse succession in tropical forests at the scale of 4 ha (total simulated area 100 ha). As a first step, we analyse the relationships between biomass and GPP, NPP and *τ* (figure 3). Each point represents a forest with a given state (includes biomass, productivity or *τ*, and the year of succession).

The relationships between biomass and the target variables are varying in the first 100 years. In the last 100 years, when the forest reaches its equilibrium state, the forest states accumulate in a cloud of points (dark blue colour). The fastest increase of target variable with increasing biomass is seen in the biomass–GPP relationship (figure 3*c* similar to figure 1). After this phase, the GPP remains stable but biomass still increases until GPP and biomass decrease and forests reach their equilibrium (point cloud in dark blue). The biomass–NPP (figure 3*b*) and biomass–*τ* (figure 3*c*) relationships over time show a smaller increase and shorter stagnation of NPP as well as *τ*. In the biomass–*τ* relationship, *τ* continues to increase despite decreasing biomass in the late successional phase, in contrast to the other cases. The highest correlation between biomass and the target variables is found in the biomass–NPP relationship (*R*^2^ = 0.71). No linear relationship can be found between GPP and biomass over time (*R*^2^ = 0.035).

**Figure 4.**
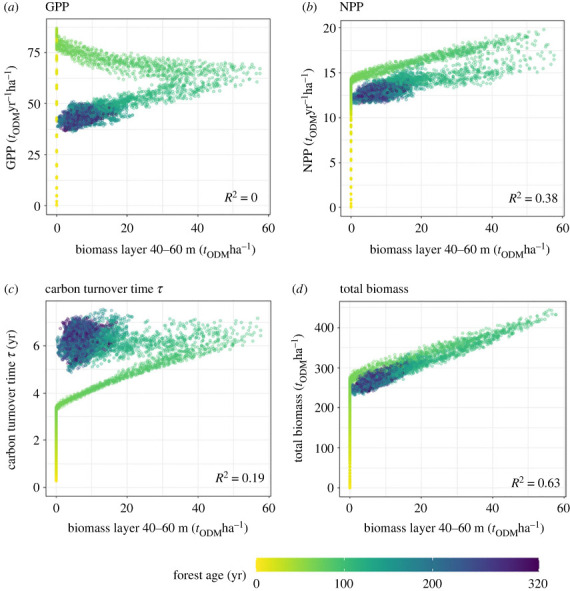
Relationship between the biomass in the height layer 40–60 m (with Δ*h* = 20 m) and (*a*) GPP, (*b*) NPP and (*c*) *τ* and (*d*) total biomass over time. The results are derived from forest simulations (tropical forest, see §2.1). Each point represents a 4 ha forest stand at a certain age (between 0 and 320 years indicated by colour). Additionally, the *R*^2^ value is calculated (right bottom corner of each graph).

In a second step, we examine how relationships change when we analyse biomass in different height layers (figure 4, here at the 40–60 m height layer).

In all four cases, we observe a strong increase GPP, NPP and *τ* in the first years of the simulation. Forest states in equilibrium state are found in all graphs (see also figure 3) after a dynamic phase of the relationships (0–about 150 years). After about 70 years, when the first trees have reached the top layer, the relationships with NPP (figure 4*b*), *τ* (figure 4*c*) and total biomass (figure 4*d*) show a phase of linear increase. After this phase, NPP and total biomass decrease with decreasing biomass in the upper forest canopy (40–60 m). *τ*, on the other hand, remains stable despite decreasing biomass in the upper forest canopy. GPP (figure 4*a*) increases very rapidly and then decreases steadily until it reaches equilibrium. The strongest correlation is found in the relationship between total biomass and biomass in the upper forest canopy (*R*^2^ = 0.63). Between the NPP and the biomass in the upper layer we find still an *R*^2^ of 0.39.

### 3.2. Estimation of GPP, NPP and *τ* with boosted regression trees using biomass information at different resolution

After we explored the relationship of the forest biomass (in one height layer) with productivity and *τ* (figure 4), we wanted to investigate the information of biomass distribution over several height layers. We tested how a BRT can use this information and which influence the spatial and vertical resolution have on the estimations. Here we used a BRT (more information in §2.3) to predict GPP, NPP and *τ* from the biomass distribution over height with different discretization (with Δ*h* of 20 m, 10 m and 2 m). Additionally, we vary the spatial resolution of the analysed forest stands (4 ha, 1 ha and 0.04 ha) to find out which cases (spatial area and Δ*h*) provide good conditions for predicting GPP, NPP and *τ*.

**Figure 5.**
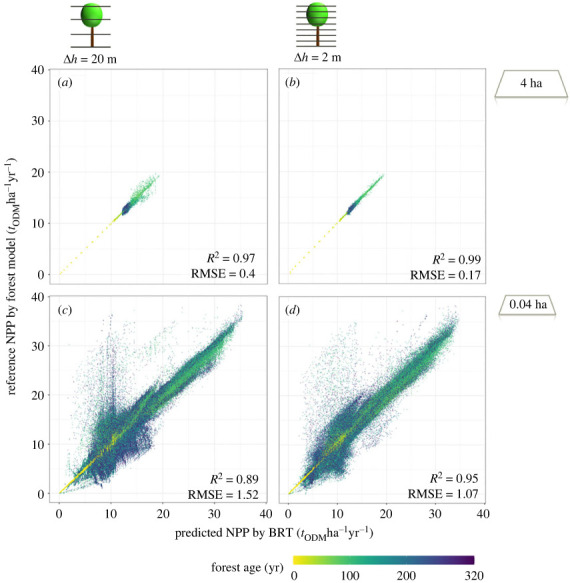
Comparison of predicted NPP derived from the BRT and reference NPP derived from a forest model. The predicted NPP is derived from the vertical biomass distributions in the simulated forests (in the height layers Δ*h* = 20 m and Δ*h* = 2 m). Each point represents a results for (*a*,*b*) a 4 ha forest and (*c*,*d*) a 0.04 ha forest. The forest age is indicated by the colour of points. Total simulated area was 100 ha (resulting in the different amount of points in top and bottom row). Results for GPP, *τ* and other resolutions see appendix figures 12–14.

At first, we want to show an excerpt of our analysis and describe the results of the predicted NPP with the help of the BRT and biomass information with Δ*h* = 20 m and 2 m and spatial resolution of 4 ha and 0.04 ha (figure 5). For all four cases, the predictions of NPP for forests in early succession are near to the simulated NPP (yellow points).

The result for NPP after succession phase (forests after 160 years–blue points) are strongly influenced by the spatial resolution (from 4 ha resolution to 0.04 ha resolution). For 4 ha forests (figure 5*a*,*b*), we observe a small cloud for the equilibrium state of forests (already seen in figures 3 and 4) which is getting smaller with a higher number of height layers (Δ*h* = 2). For 0.04 ha resolution (figure 5*c*,*d*), there are more data points (forest plots) over all (total simulated area 100 ha) but also more forest plots far from the 1:1 line. With higher vertical resolution (more height layers, Δ*h* = 2, figure 5*d*), there are fewer outliers and estimations for forest stands are nearer at the 1:1 line (similar effect to 4 ha forests in (b)). We observe a strong correlation for all four cases (*R*^2^ > 0.89). With higher spatial resolution the RMSE and the value range increase.

**Figure 6.**
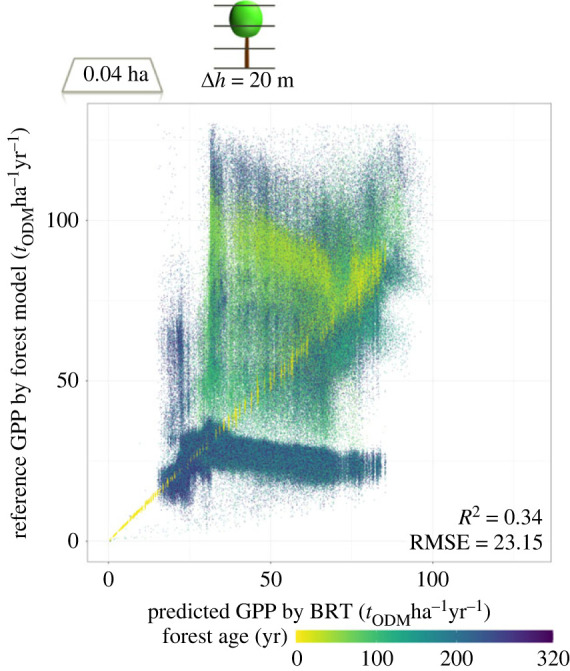
Comparison of predicted GPP derived from the BRT and reference GPP derived from a forest model. The predicted GPP is derived from the vertical biomass distributions in the simulated forests (with Δ*h* = 20 m). Each point represents a result for a 0.04 ha forest. The forest age is indicated by the colour of points. Total simulated area was 100 ha. For results for GPP, *τ* and other resolutions see appendices figures 12–14.

Comparing the estimated GPP by the BRT for 0.04 ha forests (figure 6) we observe a weaker correlation (*R*^2^ = 0.34 for Δ*h* = 20 m). We observe a strong correlation for young forest stands (yellow points near 1:1 line like in figure 5), but for forests with an age between 50 and 100 years the BRT underestimates GPP. For 0.04 ha forests with an age over 100 years we observe under- and overestimation of GPP.

**Figure 7.**
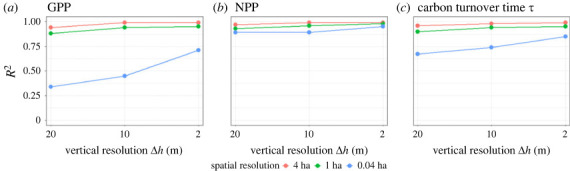
Comparison of correlation between the estimated and the reference (*a*) GPP, (*b*) NPP and (*c*) *τ*. The estimated values have been derived with a BRT using information of biomass distribution and the reference values were calculated by using the FORMIND forest model. Each point represents the *R*^2^ value of one comparison with a given spatial (indicated by colour) and vertical resolution (see figures 5 and 6 and appendix figures 12–14 on the bottom right).

Analysing a broad range of spatial resolutions for the entire forest succession of 320 years (figure 7), we got a high predictive power of structural information, as the structure-derived values correlate well with GPP and NPP of the forest. Especially for forest stands of 4 ha and 1 ha the correlation is strong for all investigated cases (*R*^2^ > 0.8). Only GPP shows weaker correlations with forest stands of 0.04 ha (details in figure 6*a*). The more information we have on the vertical biomass distribution in a forest, the better we can predict GPP, NPP and *τ*.

**Figure 8.**
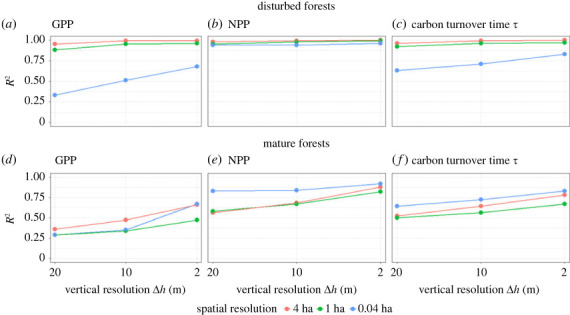
Comparison of correlation between the estimated and the reference GPP, NPP and *τ* for different types of forests ((*a*–*c*) disturbed forests; (*d*–*f*) mature forests). The estimated values have been derived with boosted regression trees using information on biomass distribution and the reference values have been obtained using a forest model. Each point represents the *R*^2^ value of one comparison with a given spatial (indicated by colour) and vertical resolution (see also appendix figures 9–11, on the bottom right).

As seen before we observed a high correlation for forest stands of 4 ha and 1 ha when training BRT with the biomass information of the whole forest succession (figure 5 and following in figure 8). In the next step, we want to analyse how the correlations change when the forest dataset is split by assuming two categories of forests: mature forests in equilibrium with an age between 160 and 320 years and disturbed forests with an age between 0 and 160 years (figure 8). We assume here that the mature stage is reached after 160 years (figure 1).

We obtain here that the high quality of the prediction with the BRT is mainly driven by the disturbed forests (figure 8*a*–*c* are similar to figure 7*a*–*c*). When analysing mature forests of different sizes, we observe a different picture. The correlations for GPP, NPP and *τ* are overall weaker (strongest *R*^2^ for NPP, weakest *R*^2^ for GPP). In contrast to the analysis of the whole forest data set (figure 7) and disturbed forests (figure 8*a*–*c*), for mature forests (figure 8*d*–*f*) the weakest correlations are obtained for forest stands of 4 ha and 1 ha size. Mature forest stands with a higher vertical resolution (Δ*h* = 10 m and 2 m) leading to better estimations of NPP, GPP and *τ*.

## 4. Discussion

### 4.1. Summary

In our study, we investigated a novel approach to estimating GPP, NPP and *τ* for a tropical forest. Here, we used an individual-based forest model to simulate forest development of a typical tropical forest in combination with a BRT to analyse the relationships between biomass distribution and GPP, NPP and *τ* at different vertical and horizontal scales. This approach was used to explore which spatial resolutions (vertical and horizontal scale) are suitable to predict forest productivity for two different forest categories (mature and disturbed forests).

When comparing simulated with predicted forest productivity we obtained surprisingly high correlations for GPP, NPP, *τ* at the scale of 4 ha and 1 ha (*R*^2^ > 0.8, figure 7). In the case of 0.04 ha scale, *τ* and especially GPP show lower correlations (figure 7*a*,*c*). These results are mainly driven by disturbed forests (results shown in figure 7*a*–*c* are similar to the results shown in figure 8*a*–*c*), which is why we split the forest data. For the prediction of productivity of mature forests, we observe lower correlations. Among them, higher correlations were achieved using biomass information from spatial scales of 0.04 ha and Δ*h* = 2 m (*R*^2^ > 0.67, figure 8*d*–*f*).

### 4.2. Obtained trends

Increased vertical resolution (especially for Δ*h* = 2 m) always leads to increasing correlations, for disturbed forests as well as mature forests (figure 8). For disturbed forests (forests younger than 160 years), the better predictions result from one-to-one relations between biomass and productivity whereas these relations show ambiguities for mature forests (forests older than 160 years). For disturbed forests, one single biomass value relates to one productivity value, whereas the occurrence of one-to-one relations disappears for forests older than 160 years (figure 3). In the case of GPP, this appearance of ambiguities already occurs for forests older than 100 years, which explains the lower correlation for GPP of disturbed forests (figure 8*a*).

The decreasing predictability of disturbed forest productivity with increasing spatial resolution (1 ha and 0.04 ha) seems counter intuitive at first glance. However, the higher the spatial resolution of biomass distribution the more ambiguities occur in relation to forest productivity and the more difficult it is to predict productivity. This becomes particularly clear for the estimation of GPP (for both categories of forests). Nevertheless, higher spatial resolution measurements (at the scale of 0.04 ha) have the potential for improved prediction quality in connection with other sources of information (e.g. with measurements of additional forest attributes by remote sensing). For example, height measurements derived from LiDAR [30] may have the potential of such synergy effects. Not only the quality of the prediction but also the categorization of forest types can be improved by additional information.

### 4.3. Limitations

Owing to the strong influence of young forests (age < 160 years) on the BRTs trained on the whole forest data, we decided to perform an additional analysis. So it turned out to be helpful to divide the training data for the BRT into different forest categories (using the age of a forest as an indicator). This partition of training data leads in our case to two different prediction possibilities (two BRTs, one for prediction of productivity of disturbed forest and one for mature forests). By partitioning the forest data by age, we assume that complex disturbed ecosystems at the scale of 4 ha can be reconstructed from the set of forest states that occur during the first 160 years of succession. It is possible that other forest landscapes occur in which disturbance patterns have caused a different biomass distribution. Future studies should investigate whether these altered disturbance distributions have an impact on prediction quality. We propose here to use thresholds of biomass values as an indicator for forest type categorizations, which are provided by the BIOMASS mission anyway.

In addition to that, our approach of categorizing forests as disturbed or mature based on forest succession is only one way to categorize forests. Different classification may yield to different results. We analysed the robustness of this approach by categorizing the forest data and in a next step the analysis could be applied to other forest category selections and forest data compositions. Our study shows that predictions from satellite data might have weaknesses for specific forest categories that may not be directly apparent. In addition, the R packages available for BRT applications vary in their quality of results and computational requirements. This leads to additional challenges for the comparability of results (see appendix tables 1–3).

### 4.4. Combining forest modeling and remote sensing

This study demonstrates how individual-based forest models can be used to explore the link between remote sensing measurements and ecosystem properties as well as forest processes. Forest models have a long tradition in analysing the impact of climate change on forest structure, species composition and biogeochemical cycles (e.g. [31–34]). As pointed out by Maréchaux *et al.* [35], forest models are also able to conduct virtual experiments beyond empirical investigations [36–38], as well as to test assumptions about ecological processes [39,40]. In addition, they can be used to reveal potential of improvements and possible gaps in knowledge as well as to guide the design of further field experiments [41,42]. Owing to increasing confidence in the ability of forest models to reflect the true behaviour of the system (e.g. due to high agreement in model comparisons—[43–45]), they are increasingly being used to investigate important relationships between different forest characteristics (e.g. [30,38,46–52]).

Studies using forest models are more and more incorporating remote sensing measurements as the amount of remote sensing data is increasing significantly [53]. For example, they derive information from remote sensing measurements about the heterogeneity of forest structure [54] or estimate carbon dynamics of forests with the use of new allometric models parametrized with tree crown data derived out of airborne measurements [55]. Forest models can be used to analyse correlations of height-related biomass measurements to above-ground carbon stocks [56]. Furthermore, forest models can derive relationships for the interpretation of remote sensing measurements, e.g. between forest height and carbon stock [30]. In addition, they can be used to create virtual remote sensing data by combining them with forward modelling of radiative transfer or LiDAR to link measurements directly to forests [16,57].

Our study can be used (i) to explore general relationships between forest properties (here for biomass and NPP and possibly for other forest properties), (ii) to explore the potential of satellite measurements (here for the BIOMASS mission) as well as (iii) to combine remote sensing, field data and modeling knowledge (e.g. using inventories or airborne forest measurements in combination with remote sensing measurements on different spatial scales). For missions that are well advanced in planning, such as the BIOMASS satellite mission, the approach provides a first estimate of expected outcomes to derive estimates of forest productivity and *τ.* Future missions may therefore benefit from similar approaches (with respect to improvements in vertical or spatial resolution) in the trade-off analysis of technical improvements.

### 4.5. Advantages of the presented approach

In this study, virtual measurements from future satellite missions are used to derive forest attributes (here GPP, NPP, *τ*). Hence, this study can be seen as an example, which shows that the newest generation of forest models provides options to further develop Level 3 satellite products. The method provides good results (e.g. *R*^2^ > 0.8 for NPP predictions see figure 7*b*) for the majority of investigated cases, in particular it provides also good results for predictions using high resolution measurements (especially for mature forests with *R*^2^ > 0.7, figure 8*e*). This is promising and shows the potential of this novel method for estimating forest productivity from the spatial distribution of biomass within a forest. One highlight of our study is that we provide a method for predicting *τ*, as other observation- and modelling-based methods are characterized by large uncertainties [58]. In addition, our approach takes advantage of individual-based models, which allow us to study forest structure in detail and relate it to a target variable—here forest productivity. By contrast, global vegetation models have a focus on large spatial scales, whereas individual-based models focus on smaller scales, as they consider processes at tree level and can thus also analyse structural dynamics of forests [35]. This allows the identification of structural properties and functional characteristics of forest ecosystems at different spatial scales.

An important result of our study is to demonstrate the potential of high-resolution tomographic satellite measurements of biomass in estimating GPP, NPP and *τ*. The developed approach enables the estimation of forest productivity based on Radar remote sensing measurements. Runing *et al.* [10] provide estimations of GPP and NPP (with resolution of 25 ha) by using NDVI and FPAR (Fraction of Absorbed Photosynthetic Active Radiation) from MODIS satellite products in combination with additional models for the estimation of maintenance respiration (in the case of NPP). The quality of these estimates varies for different tropical regions [15,59–61]. In addition, BIOMASS measurements at long wavelengths in the P-band have the advantage of avoiding saturation effects.

### 4.6. Outlook

The developed framework, which uses machine learning techniques in combination with an individual-based forest model to derive forest relationships, can also be applied to other satellite missions (beyond radar). It is able to support satellite missions in exploring the potential of different spatial resolutions (horizontal and vertical) and the relationships between observation patterns and target variables of forests. Also the combination of different measurements (inventory data or other satellite measurements) can be analysed with the presented mechanistic framework. In this way, the framework presented here might also be of interest to remote sensing platforms such as the ESA-NASA Joint Multi-Mission Algorithm and Analysis Platform (MAAP, [62]). The platform is a collaborative project focused on improving the understanding of above-ground terrestrial carbon dynamics by sharing data, scientific algorithms and computing power to support and accelerate scientific research.

The used forest model FORMIND is also able to connect forest state and structure to top of canopy reflection [57] or LiDAR measurements [16]. In this study, we have analysed a typical tropical forest and a possible next step is to apply this approach to other tropical forests. Rödig *et al.* [52] applied FORMIND in combination with space-borne LiDAR measurements to the whole Amazon, and it would be possible to use this framework in combination with our approach. We expect similar trends with different values of GPP and NPP due to climate variations. Furthermore, it is also possible to apply the approach to temperate and boreal forests [48,57,63] where we expect even stronger variations in GPP and NPP due to shorter growing seasons. Henniger *et al.* use the forest factory approach [47,64] to generate virtual forests instead of simulating forest development over time. This approach generates virtual forests with a forest generator that allows the analysis of vast virtual forest data (700 000 forest stands) for different eco regions, including forest conditions beyond those found in forest inventories. This approach is applied to a variety of other regions around the globe [64]. Combining the forest factory approach with the presented here framework could help to generalize our results on what resolutions are appropriate for predicting different forest attributes (e.g. biomass and NEE).

## 5. Conclusion

This study presents a novel approach to analyse Radar remote sensing measurements to predict biomass and productivity at different spatial scales. It shows that structural details of forests facilitate a meaningful estimation of forest productivity. Furthermore, the results demonstrate the influence of spatial resolution with differences between disturbed and mature forests. Predictions for mature forests benefit from higher spatial resolutions, whereas the prediction quality for disturbed forests decreases with higher horizontal resolution and increases with higher vertical resolution.

Overall, this study highlights the role of spatial resolution in the analysis and emphasizes the need to consider both horizontal and vertical resolution when studying the relationship between biomass distribution and productivity in forests. It also shows that forest relationships do not apply equally to all forest categories. The results highlight the potential of the approach presented and, if applied to more and larger areas, can provide valuable insights, ultimately contributing to a better understanding of forest ecosystems and more informed decision making.

## Figures and Tables

**Figure 9.**
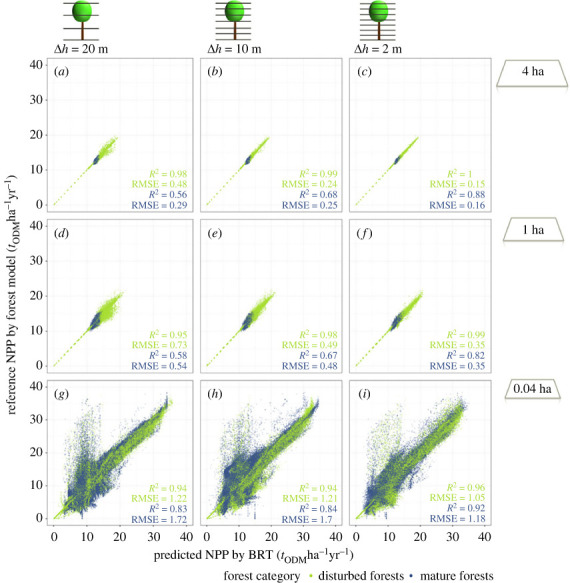
Comparison of predicted NPP derived from the BRT and reference NPP derived from simulations using the forest model FORMIND. The comparison is done for disturbed forests (green) and natural forests (blue). The predicted NPP from biomass measurements is derived from the simulated vertical and horizontal biomass distributions (descriptions above and on the right side). Each point represents a result for a forest. Total simulated area was 100 ha (resulting in the different amount of points in the rows).

**Figure 10.**
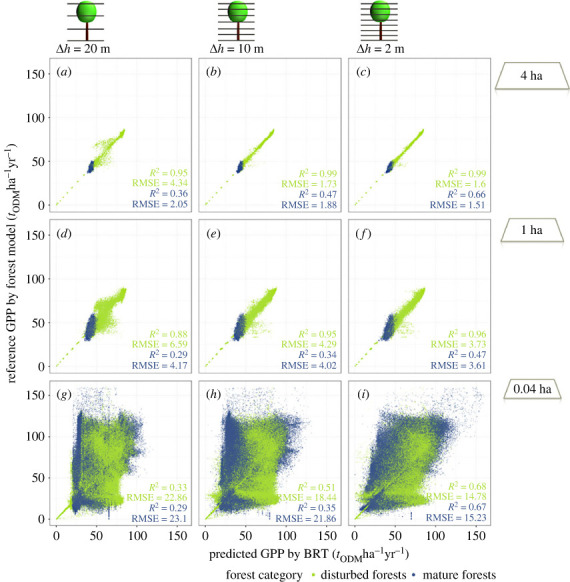
Comparison of predicted GPP derived from the BRT and reference GPP derived from simulations using the forest model FORMIND. The comparison is done for disturbed forests (green) and natural forests (blue). The predicted GPP from biomass measurements is derived from the simulated vertical and horizontal biomass distributions (descriptions above and on the right side). Each point represents a result for a forest. Total simulated area was 100 ha (resulting in the different amount of points in the rows).

**Figure 11.**
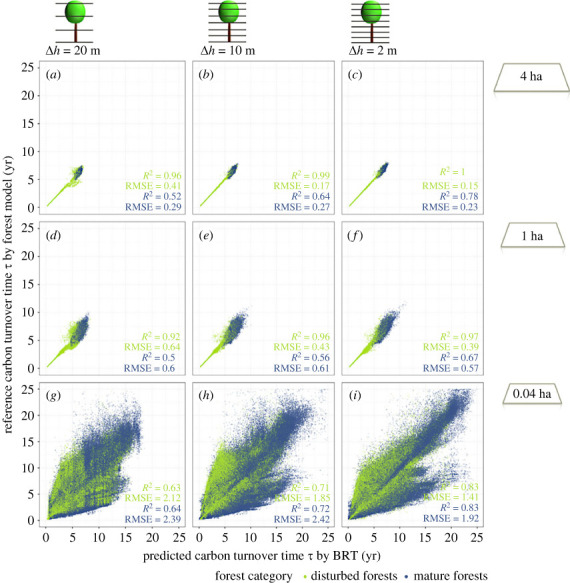
Comparison of predicted *τ* derived from the BRT and reference *τ* derived from simulations using the forest model FORMIND. The comparison is done for disturbed forests (green) and natural forests (blue). The predicted *τ* from biomass measurements is derived from the simulated vertical and horizontal biomass distributions (descriptions above and on the right side). Each point represents a result for a forest. Total simulated area was 100 ha (resulting in the different amount of points in the rows).

**Figure 12.**
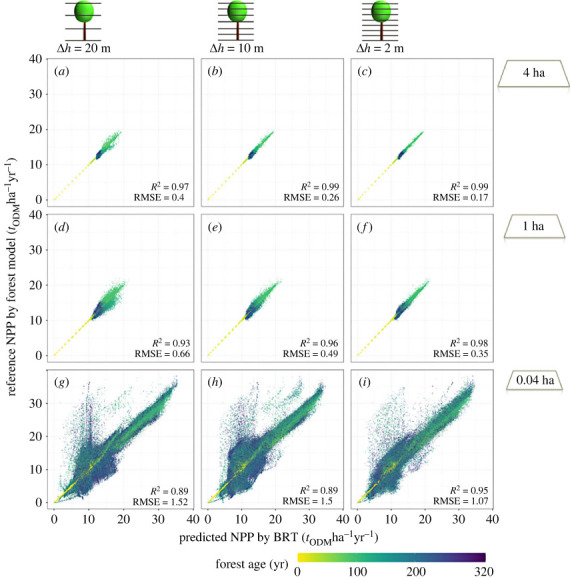
Comparison of predicted NPP derived from the BRT and reference NPP derived from simulations using the forest model FORMIND. The comparison is done for the whole forest data. The predicted NPP from biomass measurements is derived from the simulated vertical and horizontal biomass distributions (descriptions above and on the right side). Each point represents a results for a forest with a certain age (colour of points). Total simulated area was 100 ha (resulting in the different amount of points in the rows).

**Figure 13.**
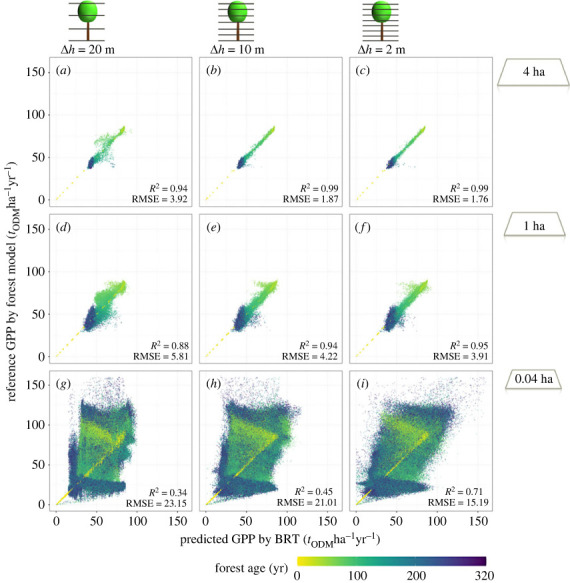
Comparison of predicted GPP derived from the BRT and reference GPP derived from simulations using the forest model FORMIND. The comparison is done for the whole forest data. The predicted GPP from biomass measurements is derived from the simulated vertical and horizontal biomass distributions (descriptions above and on the right side). Each point represents a result for a forest with a certain age (colour of points). Total simulated area was 100 ha (resulting in the different amount of points in the rows).

**Figure 14.**
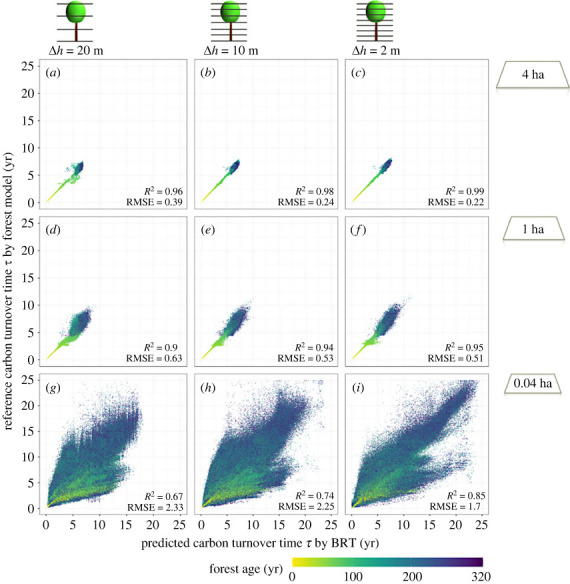
Comparison of predicted *τ* derived from the BRT and reference *τ* derived from simulations using the forest model FORMIND. The comparison is done for the whole forest data. The predicted *τ* from biomass measurements is derived from the simulated vertical and horizontal biomass distributions (descriptions above and on the right side). Each point represents a result for a forest with a certain age (colour of points). Total simulated area was 100 ha (resulting in the different amount of points in the rows).

**Figure 15.**
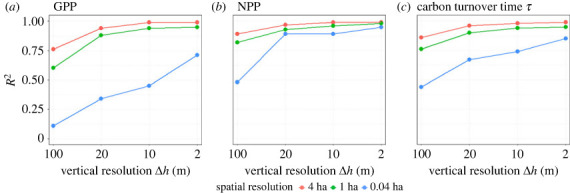
Comparison of correlation between the estimated and the reference (*a*) GPP, (*b*) NPP and (*c*) *τ*. The estimation was done with boosted regression trees using information on biomass distribution and the calculation was done with the FORMIND forest model. Each point represents the *R*^2^ value of one comparison with a given spatial (indicated by colour) and vertical resolution. Here we include also an analysis with no height layer (Δ*h* = 100 m, compared to figure 7 in the main text).

**Figure 16.**
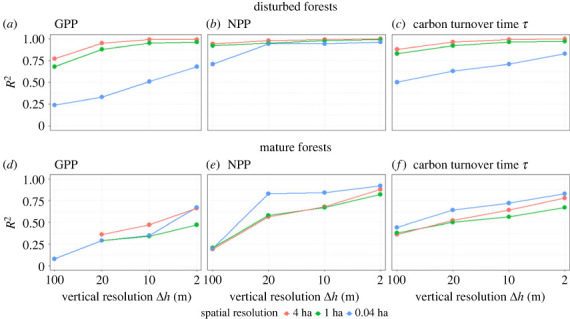
Comparison of correlation between the estimated and the reference GPP, NPP and *τ* for different types of forests ((*a*–*c*) disturbed forests; (*d*–*f*) mature forests). The estimation was done with boosted regression trees using information on biomass distribution and the calculation was done with the FORMIND forest model. Each point represents the *R*^2^ value of one comparison with a given spatial (indicated by colour) and vertical resolution. Here we include also an analysis with no height layer (Δ*h* = 100 m, compared to figure 8 in the main text).

## Data Availability

The supporting method and dataset has been updloaded here: http://doi.org/10.5281/zenodo.8239630 [65].

## Appendix A

**Table 1.** Parameters of the derived BRTs for the whole forest data (0–320 years of simulation). Depending on different spatial resolutions (vertical and horizontal) of the biomass data we derived different BRTs. Range of settings describe all possible combinations of settings (LR, learning rate; BF, bag fraction; TC, tree complexity). Not mentioned settings use the standard values of the R package dismo 1.3–14. The amount of training data is given in relative and absolute values. Additionally, the parameters of the final BRTs for GPP, NPP and *τ* are shown (normal typesetting, italic, underlined). We also included the analysis with no height layers (vertical resolution Δ*h* = 100 m figure 15 and 16).

data used for BRT (resolution)	range of setting [(LR), (BF), (TC)]	amount of training data relative (total)	used setting (**GPP**, *NPP*, *τ*) (LR, BF, TC)
4 ha, Δ*h* = 100 m	[(0.05, 0.01, 0.005),	50%(3975)	(0.01, 0.66, 3),
	(0.3, 0.5, 0.66), (3)]		(*0.01, 0.5, 3*),
			(0.01, 0.66, 3)
4 ha, Δ*h* = 20 m	[(0.05, 0.01, 0.005),	50%(3975)	(0.05, 0.5, 3),
	(0.3, 0.5, 0.66), (3)]		(*0.05, 0.66, 3*),
			(0.05, 0.5, 3)
4 ha, Δ*h* = 10 m	[(0.05, 0.01, 0.005),	50%(3975)	(0.05, 0.66, 5),
	(0.3, 0.5, 0.66), (5)]		(*0.05, 0.66, 5*),
			(0.05, 0.5, 5)
4 ha, Δ*h* = 2 m	[(0.05, 0.01, 0.005),	50%(3975)	(0.05, 0.5, 7),
	(0.3, 0.5, 0.66), (5, 7)]		(*0.05, 0.5, 7*),
			(0.05, 0.66, 7)
1 ha, Δ*h* = 100 m	[(0.05, 0.01, 0.005),	50%(15 900)	(0.05, 0.66, 3),
	(0.3, 0.5, 0.66), (3)]		(*0.01, 0.66, 3*),
			(0.05, 0.66, 3)
1 ha, Δ*h* = 20 m	[(0.05, 0.01, 0.005),	50%(15 900)	(0.05, 0.5, 3),
	(0.3, 0.5, 0.66), (3)]		(*0.05, 0.66, 3*),
			(0.05, 0.5, 3)
1 ha, Δ*h* = 10 m	[(0.05, 0.01, 0.005),	50%(15 900)	(0.05, 0.66, 5),
	(0.3, 0.5, 0.66), (5)]		(*0.05, 0.5, 5*),
			(0.05, 0.5, 5)
1 ha, Δ*h* = 2 m	[(0.05, 0.01, 0.005),	50%(15 900)	(0.05, 0.5, 7),
	(0.3, 0.5, 0.66), (5, 7)]		(*0.05, 0.5, 7*),
			(0.05, 0.5, 7)
0.04 ha, Δ*h* = 100 m	[(0.05, 0.01, 0.005),	2%(15 900)	(0.05, 0.5, 3),
	(0.3, 0.5, 0.66), (3)]		(*0.05, 0.5, 3*),
			(0.05, 0.5, 3)
0.04 ha, Δ*h* = 20 m	[(0.05, 0.01, 0.005),	2%(15 900)	(0.05, 0.5, 3),
	(0.3, 0.5, 0.66), (3)]		(*0.05, 0.5, 3*),
			(0.05, 0.5, 3)
0.04 ha, Δ*h* = 10 m	[(0.05, 0.01, 0.005),	2%(15 900)	(0.05, 0.5, 5),
	(0.3, 0.5, 0.66), (5)]		(*0.05, 0.5, 5*),
			(0.05, 0.5, 5)
0.04 ha, Δ*h* = 2 m	[(0.05, 0.01, 0.005),	2%(15 900)	(0.05, 0.5, 7),
	(0.3, 0.5, 0.66), (5, 7)]		(*0.05, 0.5, 7*),
			(0.05, 0.5, 7)

**Table 2.** Parameters of the derived BRTs for disturbed forests (0–160 years of simulation). Depending on different spatial resolutions (vertical and horizontal) of the biomass data we derived different BRTs. Range of settings describe all possible combinations of settings (LR, learning rate; BF, bag fraction; TC, tree complexity). Not mentioned settings use the standard values of the R package dismo 1.3–14. The amount of training data is given in relative and absolute values. Additionally, the parameters of the final BRTs for GPP, NPP and *τ* are shown (normal typesetting, italic, underlined). We also included the analysis with no height layers (vertical resolution Δ*h* = 100 m figure 15 and 16).

data used for BRT (resolution)	range of setting [(LR), (BF), (TC)]	amount of training data relative (total)	used setting (**GPP**, *NPP*, *τ*) (LR, BF, TC)
4 ha, Δ*h* = 100 m	[(0.05, 0.01, 0.005),	50%(1987)	(0.05, 0.5, 3),
	(0.3, 0.5, 0.66), (3)]		(*0.01, 0.66, 3*),
			(0.05, 0.66, 3)
4 ha, Δ*h* = 20 m	[(0.05, 0.01, 0.005),	50%(1987)	(0.05, 0.5, 3),
	(0.3, 0.5, 0.66), (3)]		(*0.05, 0.66, 3*),
			(0.05, 0.66, 3)
4 ha, Δ*h* = 10 m	[(0.05, 0.01, 0.005),	50%(1987)	(0.05, 0.5, 5),
	(0.3, 0.5, 0.66), (5)]		(*0.05, 0.66, 5*),
			(0.05, 0.66, 5)
4 ha, Δ*h* = 2 m	[(0.05, 0.01, 0.005),	50%(1987)	(0.05, 0.5, 7)
	(0.3, 0.5, 0.66), (5, 7)]		(*0.05, 0.5, 7*),
			(0.05, 0.66, 7)
1 ha, Δ*h* = 100 m	[(0.05, 0.01, 0.005),	50%(7950)	(0.05, 0.66, 3),
	(0.3, 0.5, 0.66), (3)]		(*0.01, 0.66, 3*),
			(0.01, 0.5, 3)
1 ha, Δ*h* = 20 m	[(0.05, 0.01, 0.005),	50%(7950)	(0.05, 0.5, 3),
	(0.3, 0.5, 0.66), (3)]		(*0.05, 0.66, 3*),
			(0.05, 0.5, 3)
1 ha, Δ*h* = 10 m	[(0.05, 0.01, 0.005),	50%(7950)	(0.05, 0.66, 5),
	(0.3, 0.5, 0.66), (5)]		(*0.05, 0.5, 5*),
			(0.05, 0.66, 5)
1 ha, Δ*h* = 2 m	[(0.05, 0.01, 0.005),	50%(7950)	(0.05, 0.66, 7),
	(0.3, 0.5, 0.66), (5, 7)]		(*0.05, 0.5, 7*),
			(0.05, 0.5, 7)
0.04 ha, Δ*h* = 100 m	[(0.05, 0.01, 0.005),	2%(7950)	(0.01, 0.66, 3),
	(0.3, 0.5, 0.66), (3)]		(*0.05, 0.5, 3*),
			(0.01, 0.5, 3)
0.04 ha, Δ*h* = 20 m	[(0.05, 0.01, 0.005),	2%(7950)	(0.05, 0.5, 3),
	(0.3, 0.5, 0.66), (3)]		(*0.05, 0.5, 3*),
			(0.05, 0.5, 3)
0.04 ha, Δ*h* = 10 m	[(0.05, 0.01, 0.005),	2%(7950)	(0.05, 0.5, 5),
	(0.3, 0.5, 0.66), (5)]		(*0.05, 0.5, 5*),
			(0.05, 0.5, 5)
0.04 ha, Δ*h* = 2 m	[(0.05, 0.01, 0.005),	2%(7950)	(0.05, 0.5, 7),
	(0.3, 0.5, 0.66), (5, 7)]		(*0.05, 0.5, 7*),
			(0.05, 0.5, 7)

**Table 3.** Parameters of the derived BRTs for mature forests (160–320 years of simulation). Depending on different spatial resolutions (vertical and horizontal) of the biomass data we derived different BRTs. Range of settings describe all possible combinations of settings (LR, learning rate; BF, bag fraction; TC, tree complexity). Not mentioned settings use the standard values of the R package dismo 1.3–14. The amount of training data is given in relative and absolute values. Additionally, the parameters of the final BRTs for GPP, NPP and *τ* are shown (normal typesetting, italic, underlined). We also included the analysis with no height layers (vertical resolution Δ*h* = 100 m figures 15 and 16). Please note, the R package did not find suitable parameters for the prediction of GPP in two cases (even with smaller step size and smaller learning rate).

data used for BRT (resolution)	range of setting [(LR), (BF), (TC)]	amount of training data relative (total)	used setting (**GPP**, *NPP*, *τ*) (LR, BF, TC)
4 ha, Δ*h* = 100 m	[(0.05, 0.01, 0.005),	50%(1987)	−,
	(0.3, 0.5, 0.66), (3)]		(*0.01, 0.5, 3*),
			(0.01, 0.66, 3)
4 ha, Δ*h* = 20 m	[(0.05, 0.01, 0.005),	50%(1987)	(0.05, 0.66, 3),
	(0.3, 0.5, 0.66), (3)]		(*0.01, 0.66, 3*),
			(0.05, 0.66, 3)
4 ha, Δ*h* = 10 m	[(0.05, 0.01, 0.005),	50%(1987)	(0.05, 0.66, 5),
	(0.3, 0.5, 0.66), (5)]		(*0.05, 0.66, 5*),
			(0.05, 0.5, 5)
4 ha, Δ*h* = 2 m	[(0.05, 0.01, 0.005),	50%(1987)	(0.05, 0.66, 7),
	(0.3, 0.5, 0.66), (5, 7)]		(*0.05, 0.66, 7*),
			(0.05, 0.5, 7)
1 ha, Δ*h* = 100 m	[(0.05, 0.01, 0.005),	50%(7950)	−,
	(0.3, 0.5, 0.66), (3)]		(*0.01, 0.66, 3*),
			(0.01, 0.5, 3)
1 ha, Δ*h* = 20 m	[(0.05, 0.01, 0.005),	50%(7950)	(0.05, 0.66, 3),
	(0.3, 0.5, 0.66), (3)]		(*0.05, 0.66, 3*),
			(0.05, 0.66, 3)
1 ha, Δ*h* = 10 m	[(0.05, 0.01, 0.005),	50%(7950)	(0.05, 0.66, 5),
	(0.3, 0.5, 0.66), (5)]		(*0.05, 0.66, 5*),
			(0.05, 0.66, 5)
1 ha, Δ*h* = 2 m	[(0.05, 0.01, 0.005),	50%(7950)	(0.05, 0.5, 7)
	(0.3, 0.5, 0.66), (5, 7)]		(*0.05, 0.66, 7*),
			(0.05, 0.5, 7)
0.04 ha, Δ*h* = 100 m	[(0.05, 0.01, 0.005),	2%(7950)	(0.01, 0.5, 3),
	(0.3, 0.5, 0.66), (3)]		(*0.01, 0.5, 3*),
			(0.01, 0.66, 3)
0.04 ha, Δ*h* = 20 m	[(0.05, 0.01, 0.005),	2%(7950)	(0.05, 0.66, 3),
	(0.3, 0.5, 0.66), (3)]		(*0.05, 0.66, 3*),
			(0.05, 0.5, 3)
0.04 ha, Δ*h* = 10 m	[(0.05, 0.01, 0.005),	2%(7950)	(0.05, 0.5, 5),
	(0.3, 0.5, 0.66), (5)]		(*0.05, 0.66, 5*),
			(0.05, 0.66, 5)
0.04 ha, Δ*h* = 2 m	[(0.05, 0.01, 0.005),	2%(7950)	(0.05, 0.66, 7),
	(0.3, 0.5, 0.66), (5, 7)]		(*0.05, 0.66, 7*),
			(0.05, 0.66, 7)
